# Phallus Reconstruction Using the Third Finger Transplant Method: A Case Report

**DOI:** 10.7759/cureus.69828

**Published:** 2024-09-21

**Authors:** Ketevan Kuzanov, Davit Aptsiauri, Ivane Kuzanov

**Affiliations:** 1 Department of Plastic and Reconstructive Surgery, Riga Stradins University, Riga, LVA; 2 Department of Plastic and Reconstructive Surgery, Kuzanov Clinic, Tbilisi, GEO; 3 Department of Plastic and Reconstructive Surgery, Total Charm Tbilisi, Tbilisi, GEO

**Keywords:** one-stage intervention, phallus reconstruction, radial forearm flap, reconstructive surgery, third finger flap, third finger transplant method

## Abstract

There are many methods of phallus reconstruction, often requiring the use of prosthetics and multiple-stage interventions. The unique method discussed in this case report uses a finger complex in order to create a neourethra as well as act in place of the prosthesis, thus offering a one-stage intervention with the restoration of full biomechanical functions of the phallus and no need for revision surgeries. We present a patient, a 52-year-old male, who had previously undergone a subtotal penile resection due to oncological disease. In our clinic, he then underwent a phallus reconstruction surgery using a third finger from a non-dominant hand and radial forearm flap. After the surgery, he was able to regain full biomechanical functions of the phallus within a month as well as achieve adequate tactile and erogenous sensitivity.

## Introduction

Nowadays, there are numerous methods of phallus reconstruction, and the goal of such surgical intervention is to form a fully functioning urethra, maintain tactile and erogenous sensitivity, sufficient rigidity, and restore sexual function as well as esthetic comprehensibility to both the patient and their partners. The unique method discussed in the case report offers a one-stage intervention using a finger complex in combination with either a thoracodorsal or radial flap. There is a possibility of inclusion of necessary volumes of tissues, restoration of a high level of sensitivity and it avoids penile prosthesis, thus any revision surgeries [1].

## Case presentation

**Figure 1:**
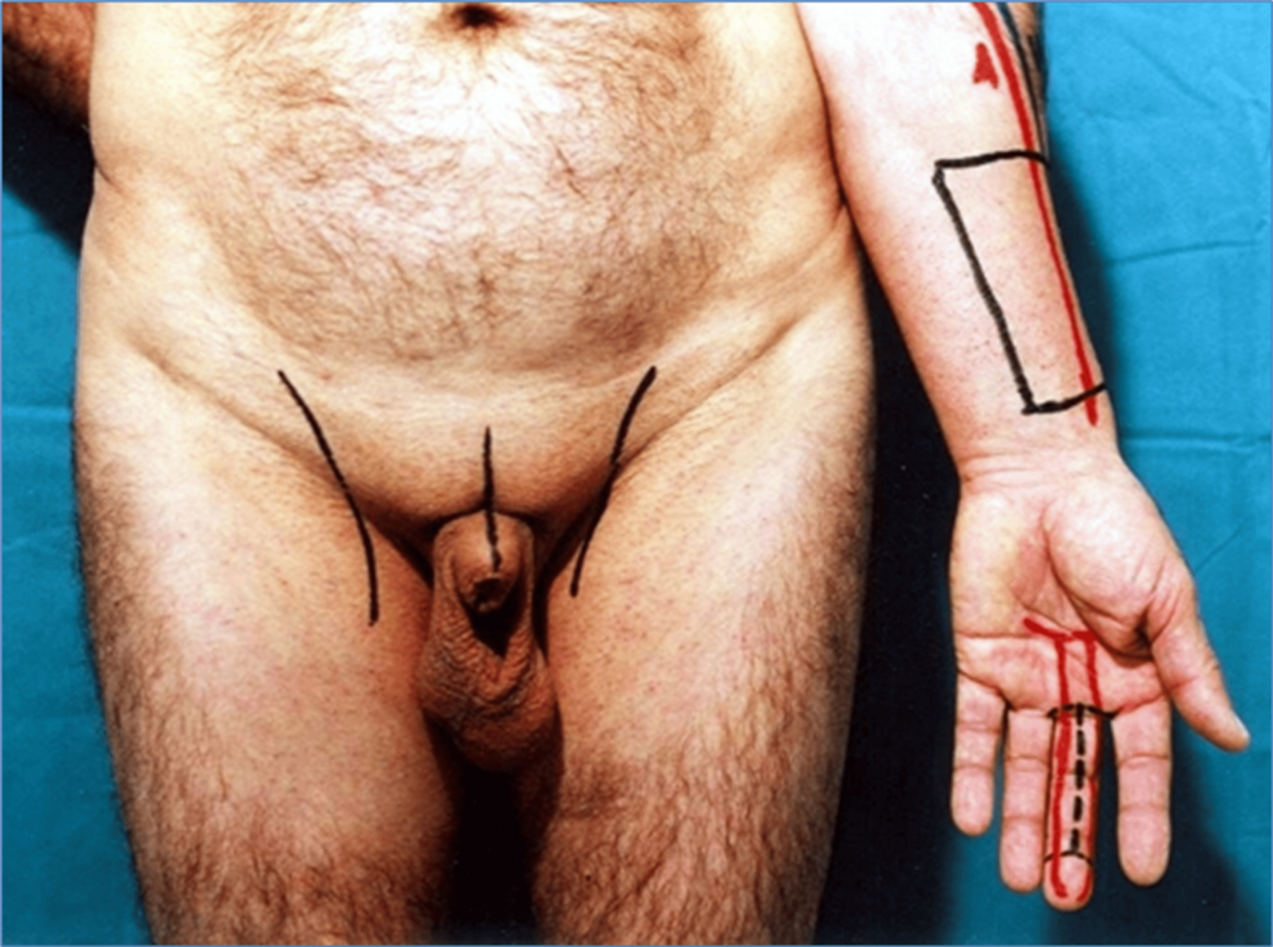
Penile stump post-oncologic penectomy.

The patient is a 52-year-old male who had undergone a penile resection surgery due to an oncological disease. The penile stump is three centimeters long. In our clinic, he underwent a phallus reconstruction surgery using a third finger from a non-dominant hand and a radial forearm flap (Figure 1).

**Figure 2:**
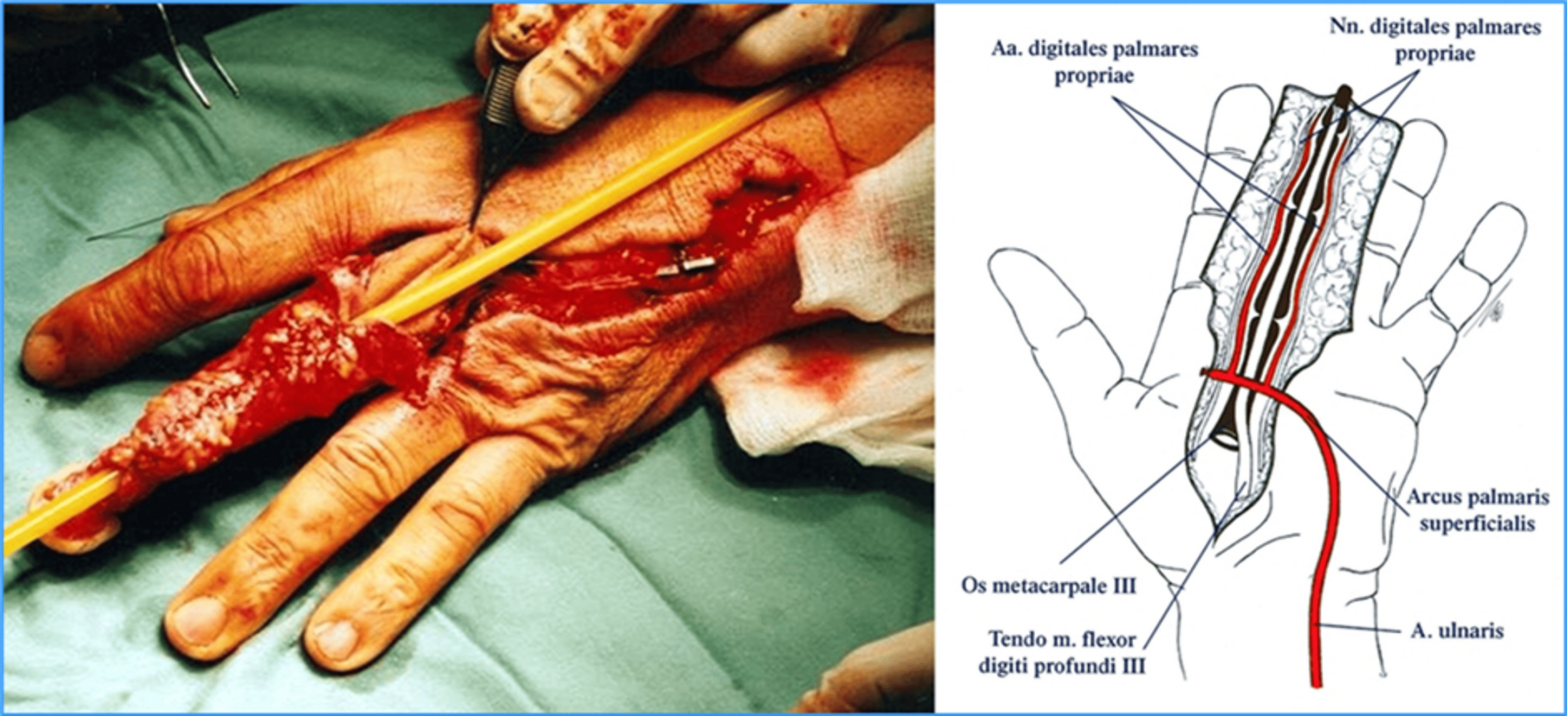
Creation of neourethra from the skin of the middle finger. Creation of Dr. Alexander Kutubidze. Property of Prof. Ivane Kuzanov.

**Figure 3:**
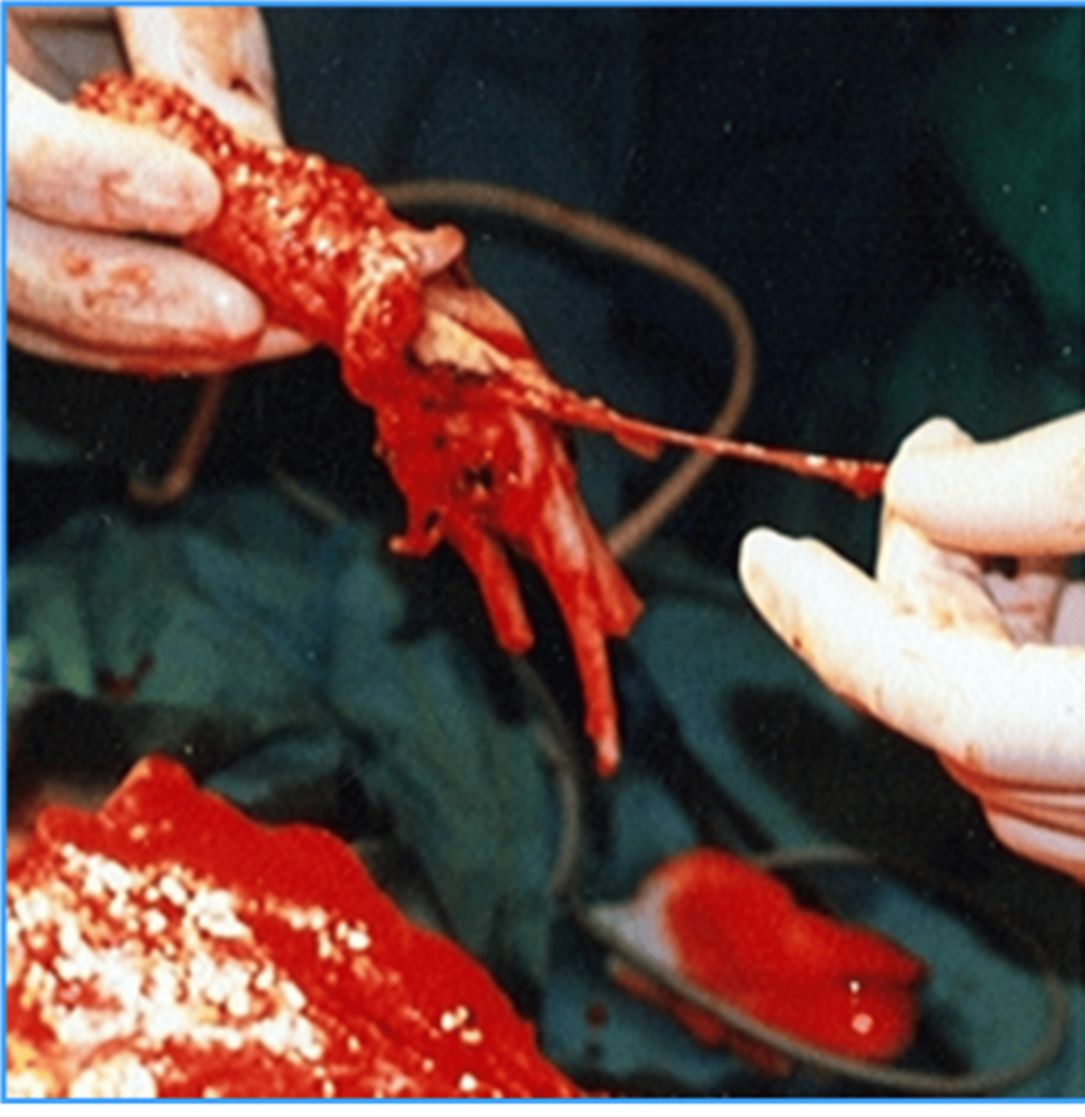
Digital flap with formed neourethra.

To create a neourethra, a longitudinal incision is made in the palmar side of the middle finger. The skin is then turned dorsally and sutured, thus creating a neourethra (Figures 2, 3).

**Figure 4:**
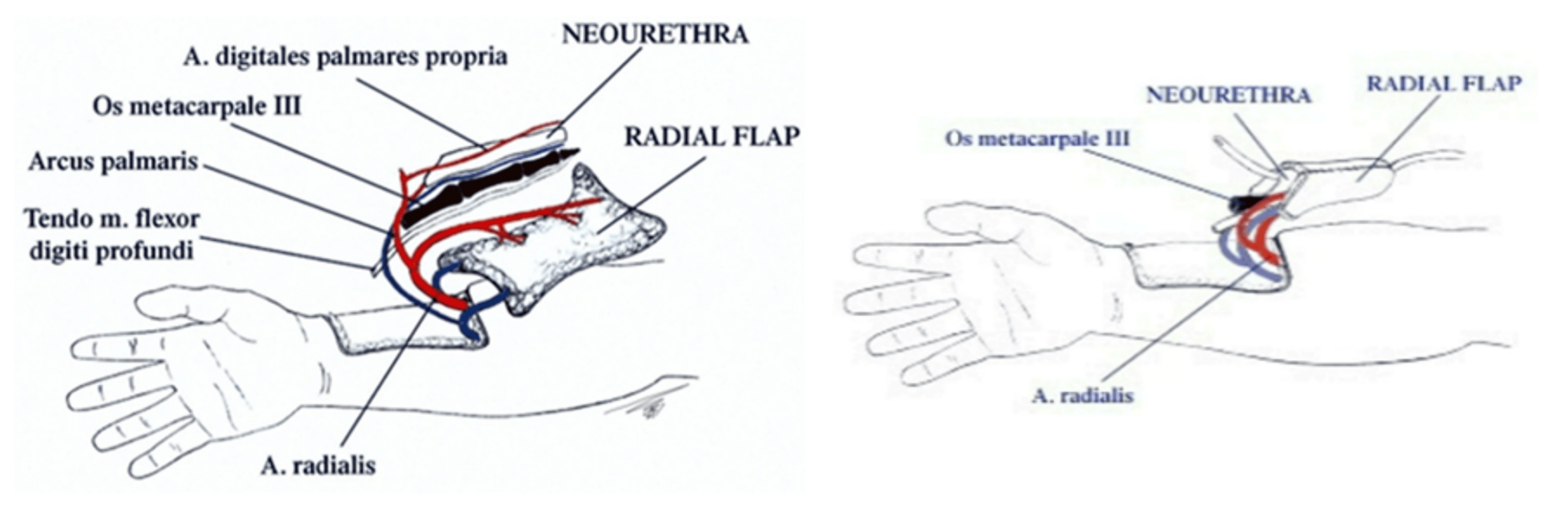
Creation of anastomosis between the digital and recipient flaps and formation of the neophallus. Creation of Dr. Alexander Kutubidze. Property of Prof. Ivane Kuzanov.

After the digital flap with a neourethra is formed, it is then transferred to a prepared radial flap zone. The end-to-side anastomosis between the arcus palmaris and radial vessels is created. The medial and lateral antebrachial cutaneous nerves are then identified and preserved (Figure 4).

**Figure 5:**
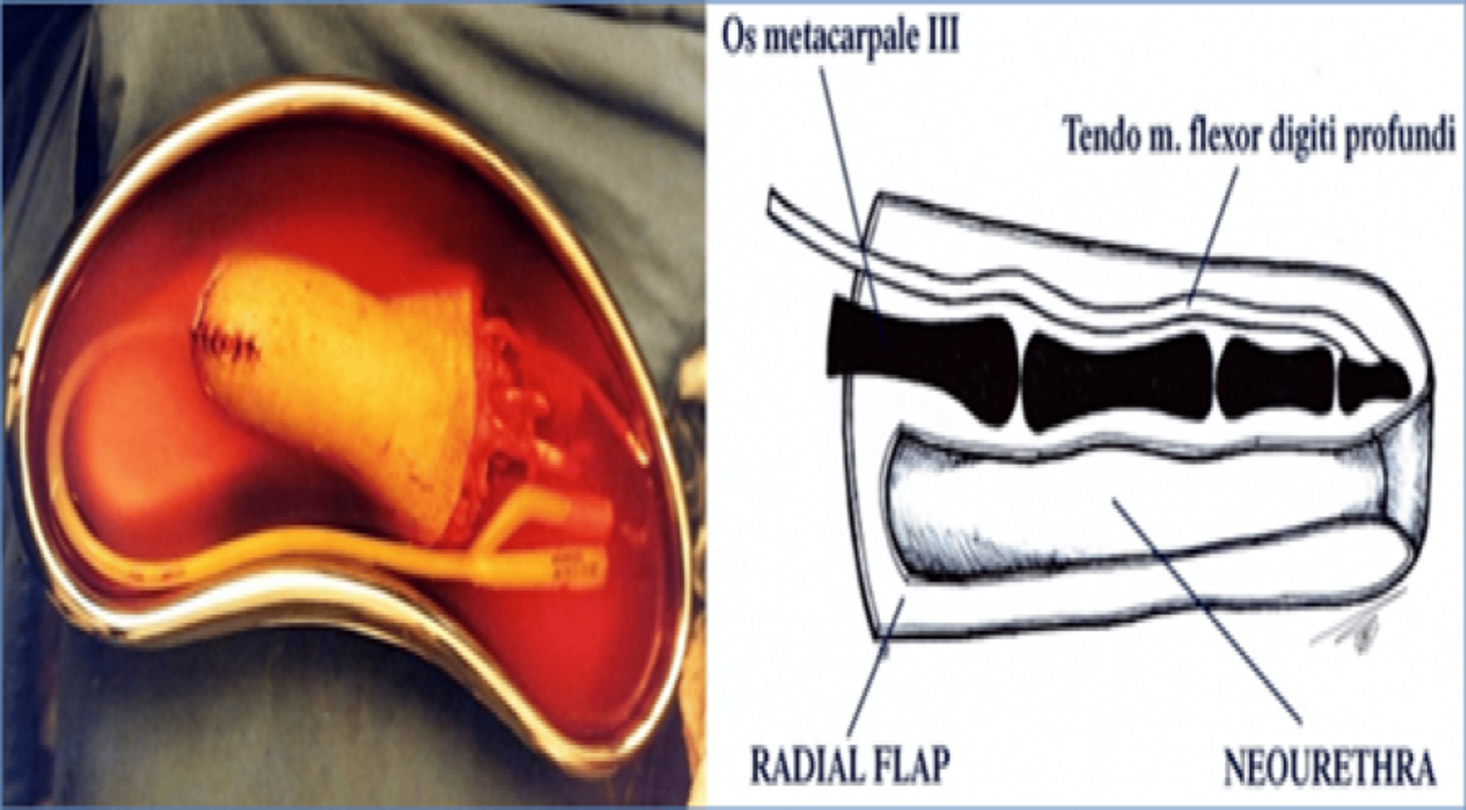
Neophallus and its anatomical structures. Creation of Dr. Alexander Kutubidze. Property of Prof. Ivane Kuzanov.

By suturing the radial forearm flap, the neophallus is formed. The finger phalanges will provide rigidity and enable erectile function, thereby replacing the penile prosthesis. The dorsally turned and sutured skin will provide the function of the neourethra (Figure 5).

**Figure 6:**
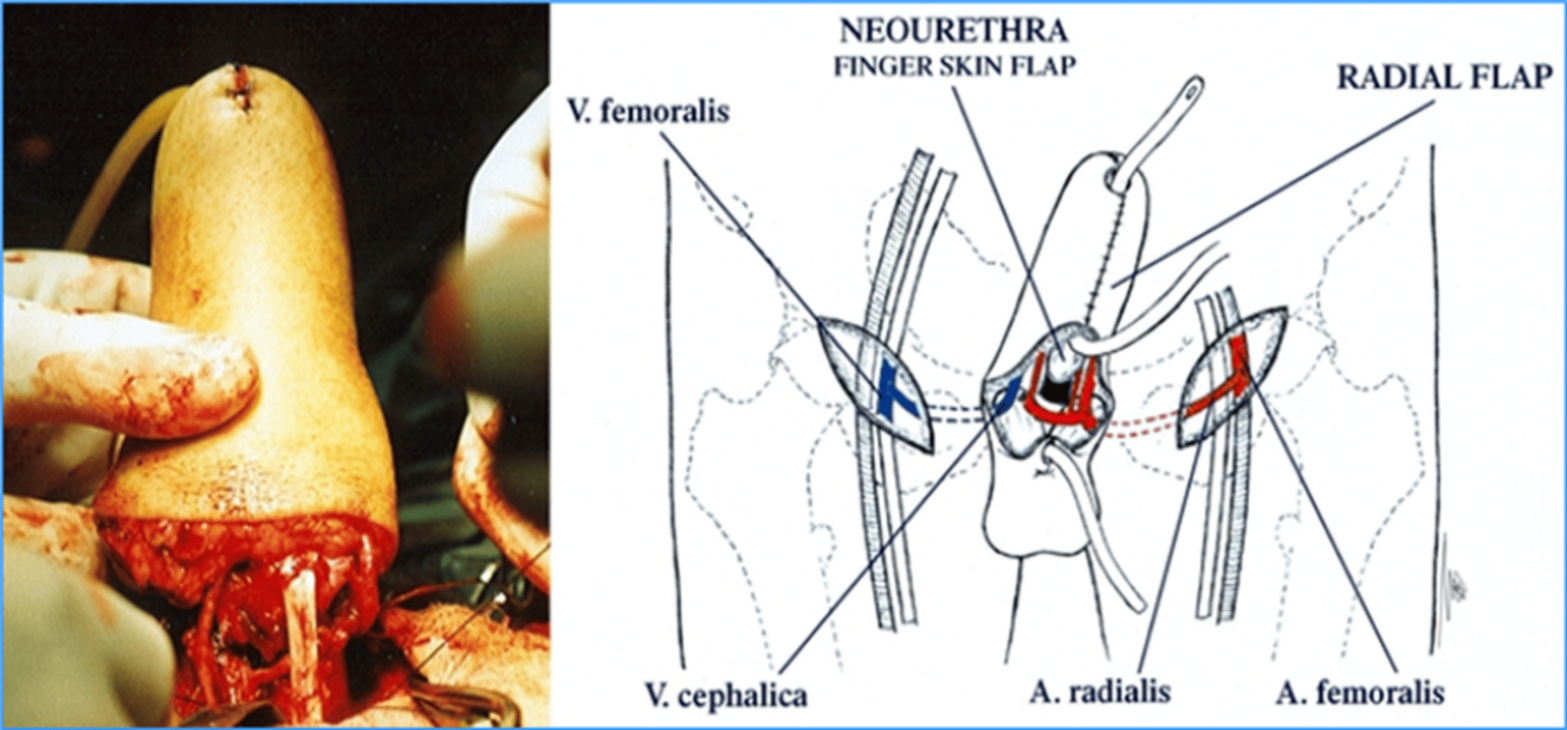
Formation of anastomosis between the neophallus and the stump. Creation of Dr. Alexander Kutubidze. Property of Prof. Ivane Kuzanov.

**Figure 7:**
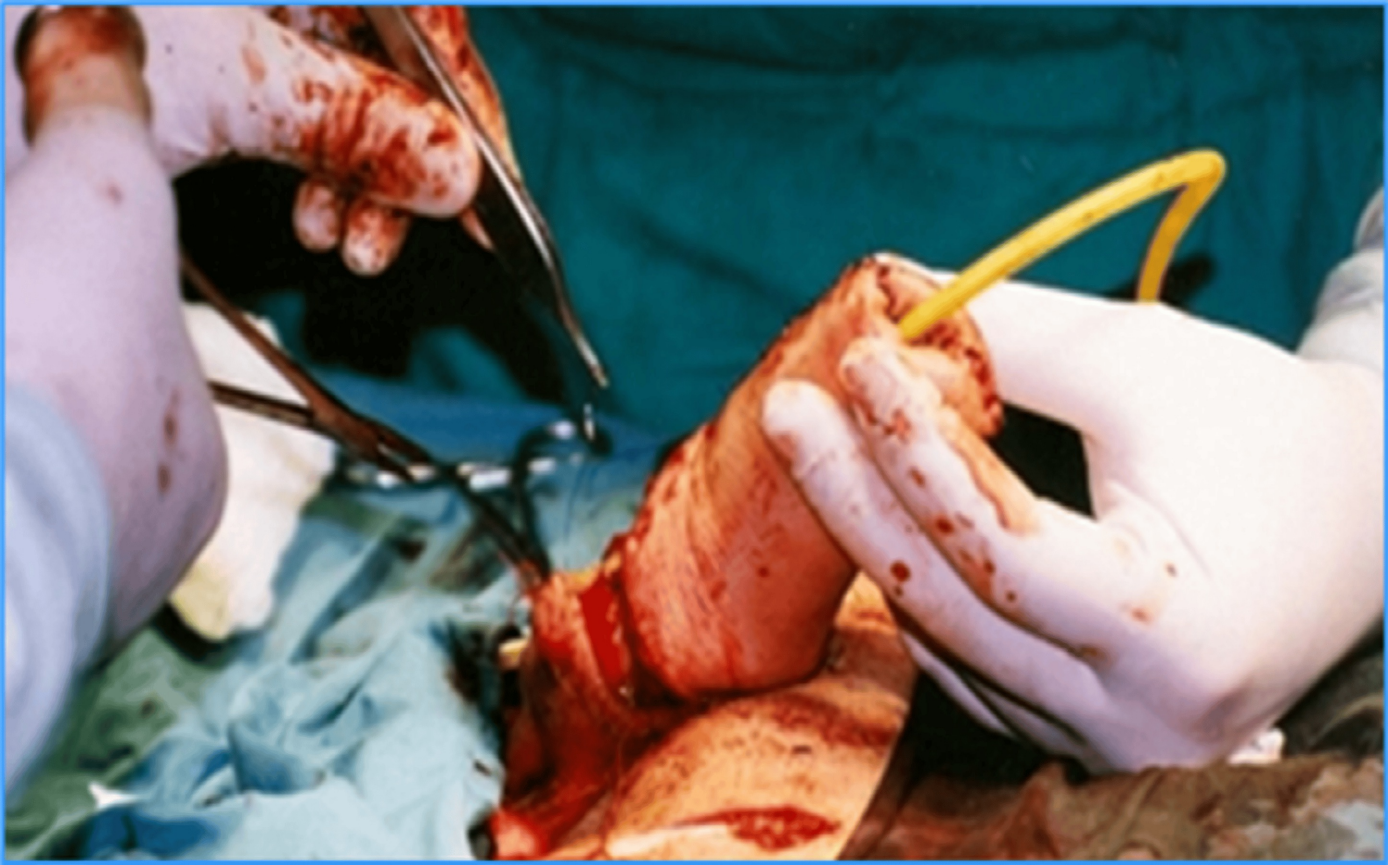
Connecting the neophallus to the stump.

The created neophallus must next be transported to the recipient zone, or the stump, where its urethra is connected to the neourethra. After that, an end-to-side anastomosis is formed between the radial and femoral vessels. The cavernous bodies of the stump are inserted into the neophallus in order to restore rigidity and help with erection. The finger phalanges are fixed to the pubic bone. For the restoration of tactile sensitivity, the lateral antebrachial cutaneous nerve is anastomosed with the ilioinguinal nerve. In order to restore the erogenous sensitivity, the medial antebrachial cutaneous nerve is anastomosed with the dorsal penile nerve (Figures 6, 7). 

**Figure 8:**
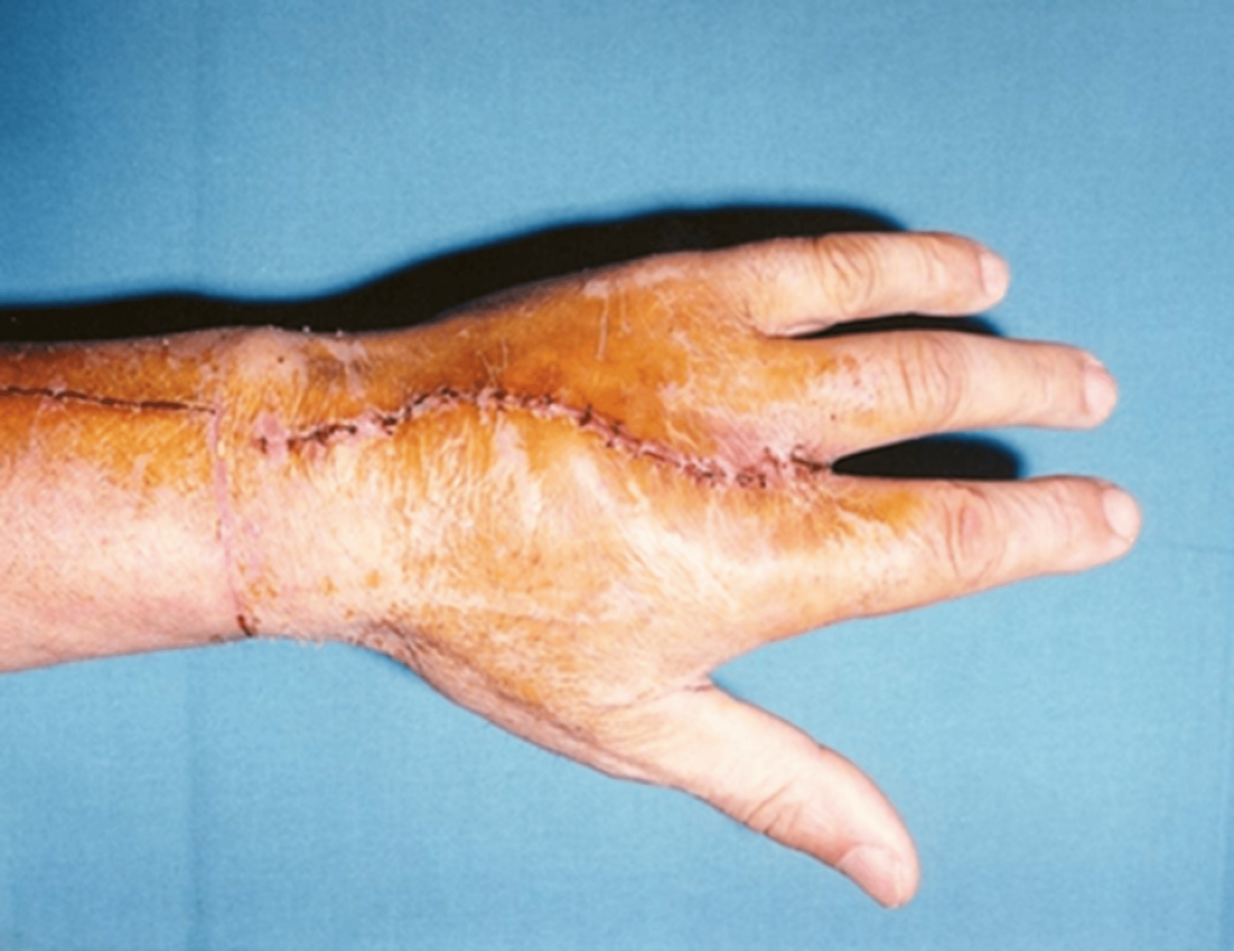
The non-dominant hand post-surgery.

**Figure 9:**
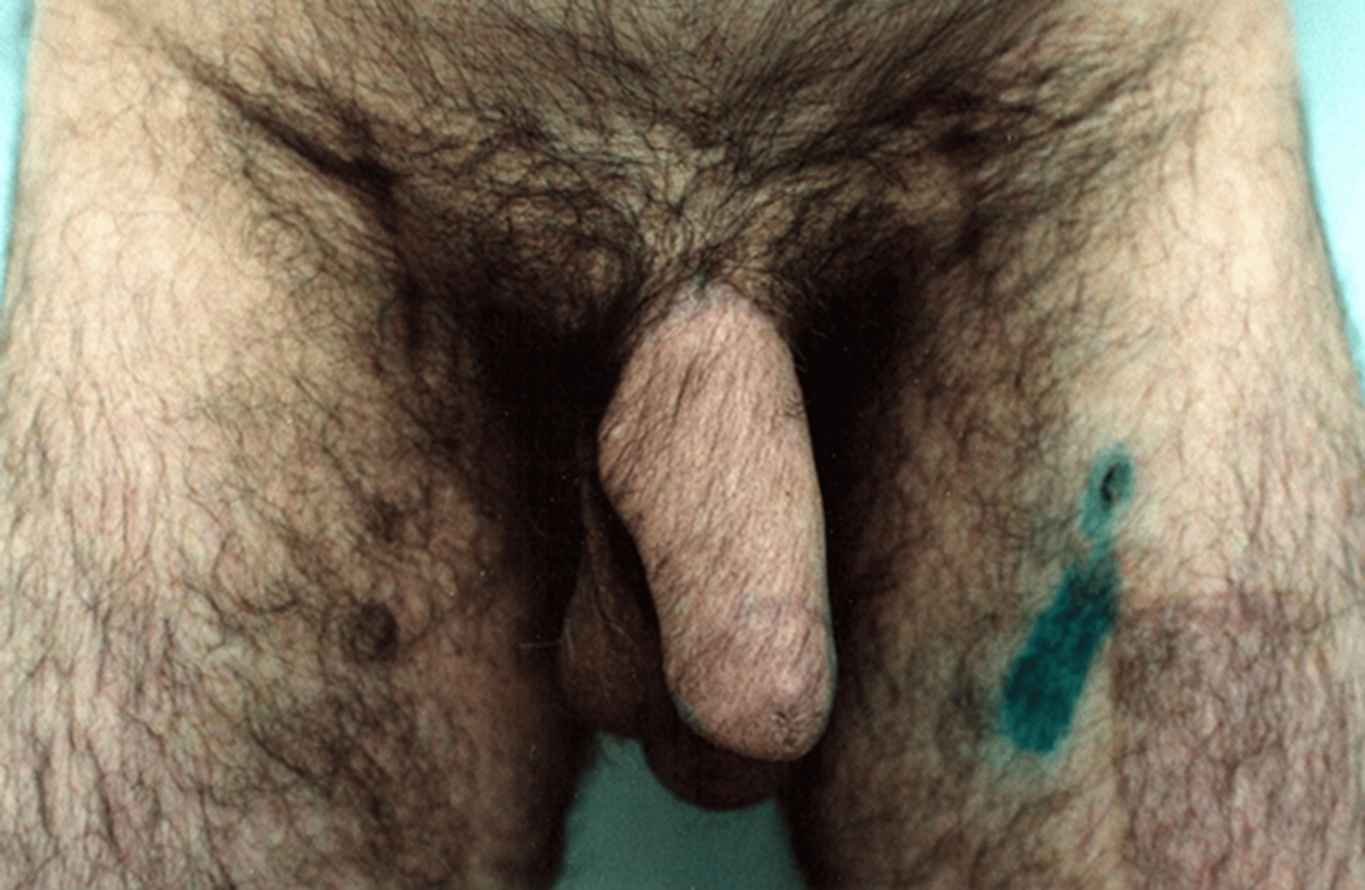
Phallus a month post-surgery.

The catheter was removed twelve days following the surgery, and the urinary function was restored. Additionally, sexual function was restored one month after the surgery. The tactile and erogenous sensitivity was regained over three months (Figures 8, 9).

**Figure 10:**
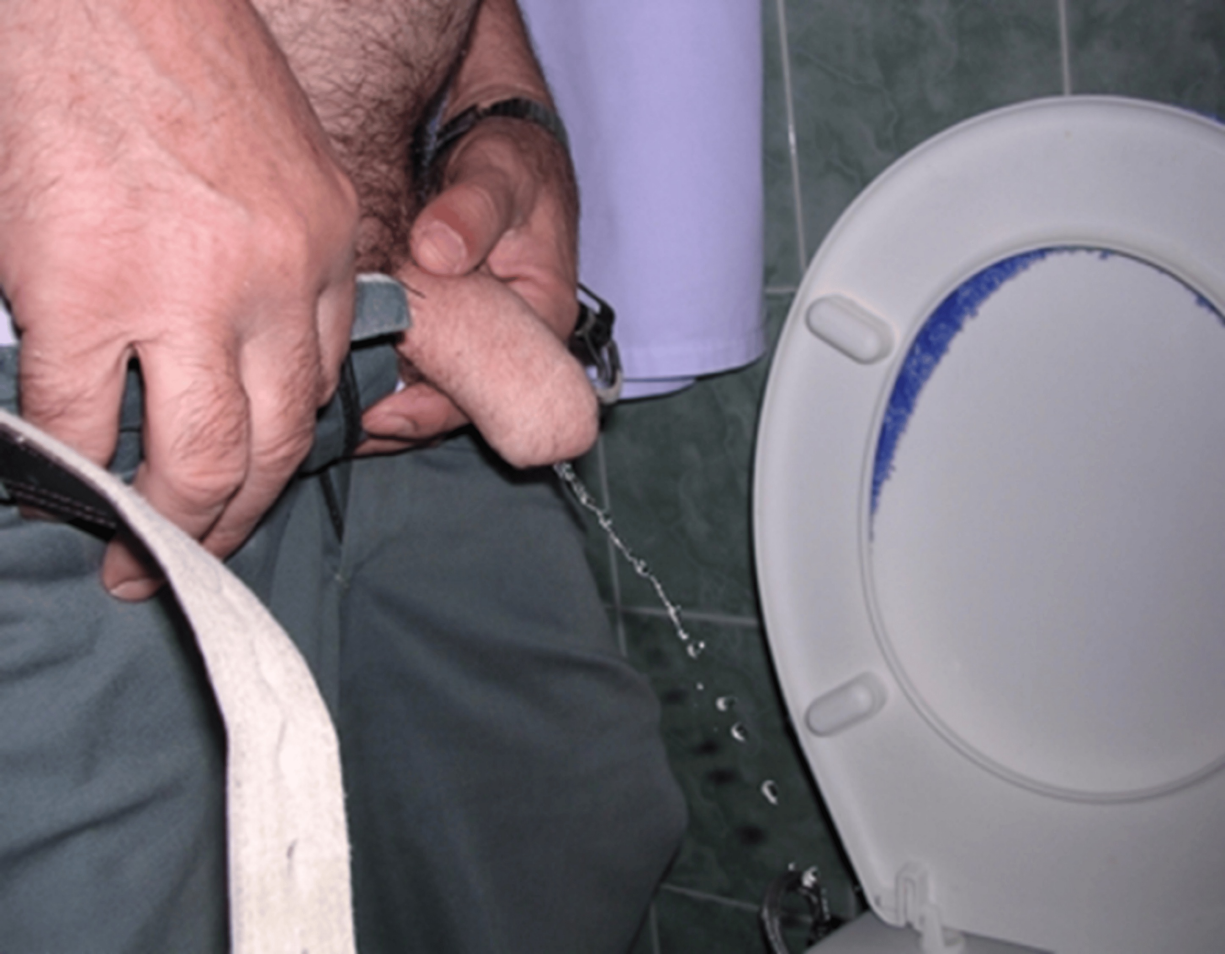
Phallus ten years post-surgery. Urinary function remained intact.

**Figure 11:**
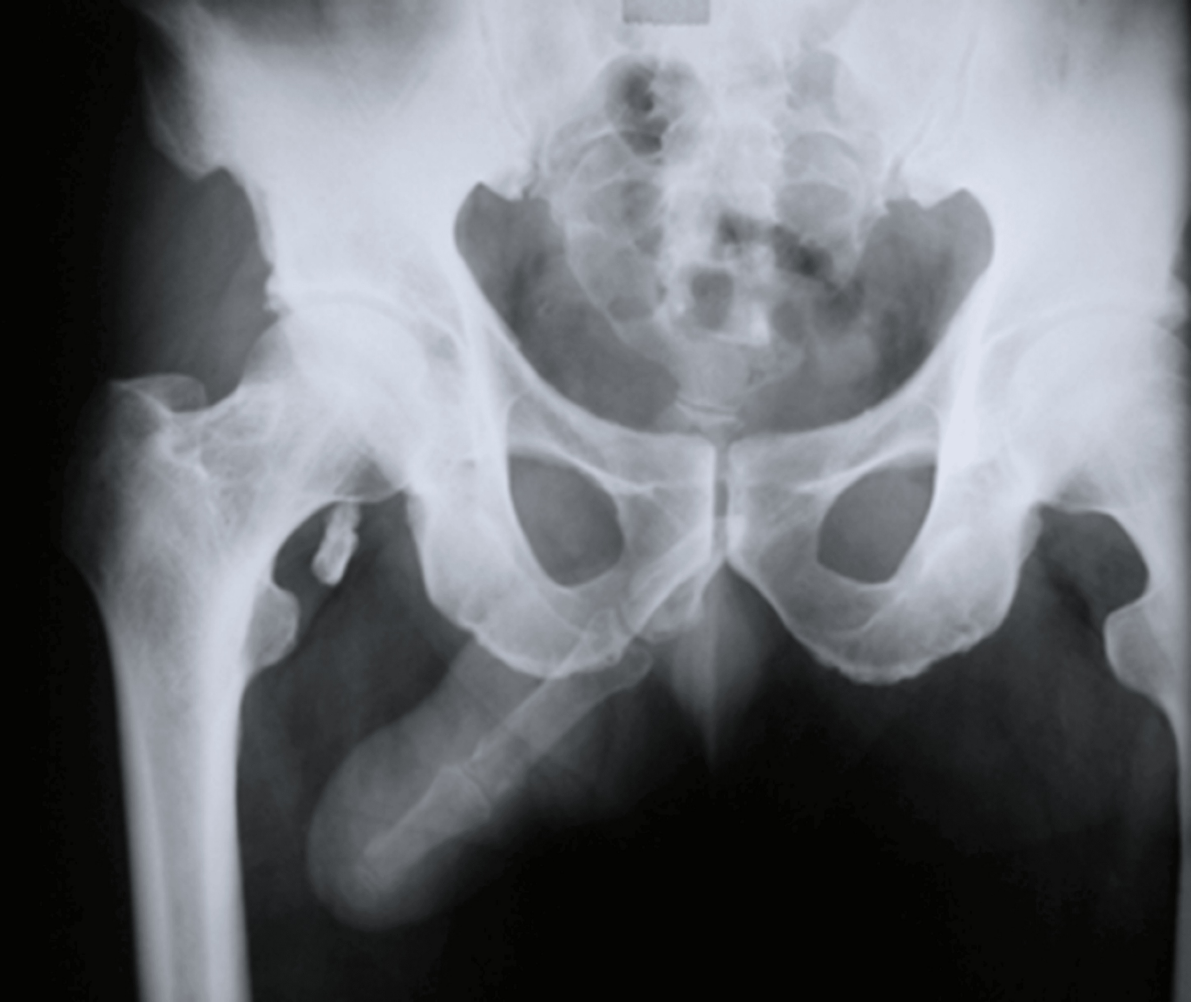
X-ray ten years post-surgery. No phalangeal bone resorption was noted, indicating an adequate blood supply.

Follow-up after ten years of the procedure confirmed that the patient's sexual and urinary functions were still unimpaired, and the X-ray indicates that there is an adequate blood supply since there were no signs of finger phalangeal resorption (Figures 10, 11).

## Discussion

Not only does the penis carry urinary and sexual functions, but it also assumes a massive psychological role in a male's life. It is widely considered to be a symbol of masculinity, fertility, strength, and male power [2]. The size and appearance can significantly impact body image and self-esteem, leading to feelings of inadequacy or satisfaction depending on beauty standards. Considering the latter, males strive to achieve the desired look even with surgical intervention. As such, we can safely say that penis plays a tremendous role in male psychological well-being. Losing a phallus for whatever reason, be it oncological, traumatic, or other, can have a devastating effect on man’s psychosocial wellness and quality of life [3], which brings us to the need for penile reconstructive surgeries.

Phalloplasty is a surgical procedure of reconstructing or creating a penis-like structure. The first successful operation was conducted in 1936 using an abdominal flap and a rib cartilage [4]. Since then, many different methods have been developed, offering various numbers of surgical interventions, recovery periods, flap locations, and so on.

The most commonly used surgical techniques require a penile implant to maintain rigidity and restore the penis' sexual function. It is important to remember that the implantation of such a penile prosthesis is typically done a year after the creation of the phallus [4]. Thus, the sexual function is restored only then. The third finger transplant method offers a one-stage intervention and uses no penile prosthesis, which allows faster recovery and restoration of the biomechanical functions of the phallus. In the scenario discussed in this case report, the patient was able to regain his sexual function only a month post-surgery.

In addition, no prosthesis means there are no complications related to them, such as prosthesis malfunction and penile corporal perforation [5].

Furthermore, by avoiding the prosthesis, the third finger transplant method also avoids the revision surgeries, which are necessary for penile prosthesis in around 30% of patients over the course of 15 years after the operation [6].

The third finger transplant method is more suitable for people looking for a technique with as little surgical intervention as possible and rapid functional recovery. This approach may be particularly intriguing to those with reservations about artificial implants.

## Conclusions

This case report demonstrates a successful one-stage phallus reconstruction utilizing a third finger and radial flap in a patient with a subtotal penectomy due to malignancy. Regardless of losing his finger, the patient was able to regain urinary and sexual functions of the phallus as well as esthetical comprehensibility and erogenous sensitivity, with only one-stage intervention and no future surgeries. This result proved to be long-term since a ten-year follow-up showed that all the biomechanical functions remained intact.
